# Experimental Investigation of Magnetic Nanoparticle-Enhanced Microwave Hyperthermia

**DOI:** 10.3390/jfb8030021

**Published:** 2017-06-22

**Authors:** Brogan T. McWilliams, Hongwang Wang, Valerie J. Binns, Sergio Curto, Stefan H. Bossmann, Punit Prakash

**Affiliations:** 1 Department of Electrical and Computer Engineering, Kansas State University, 3078 Engineering Hall, Manhattan, KS 66506, USA; mcwilliamsbrogan@gmail.com (B.T.M.); vbinns@kumc.edu (V.J.B.); s.curto@erasmusmc.nl (S.C.); 2 Department of Chemistry, Kansas State University, 213 CBC Building, Manhattan, KS 66506, USA; hongwang@ksu.edu (H.W.); sbossman@ksu.edu (S.H.B.)

**Keywords:** magnetic nanoparticles, microwave hyperthermia, microwave ablation, nanoparticle-enhanced thermal therapy

## Abstract

The objective of this study was to evaluate microwave heating enhancements offered by iron/iron oxide nanoparticles dispersed within tissue-mimicking media for improving efficacy of microwave thermal therapy. The following dopamine-coated magnetic nanoparticles (MNPs) were considered: 10 and 20 nm diameter spherical core/shell Fe/Fe_3_O_4_, 20 nm edge-length cubic Fe_3_O_4_, and 45 nm edge-length/10 nm height hexagonal Fe_3_O_4_. Microwave heating enhancements were experimentally measured with MNPs dissolved in an agar phantom, placed within a rectangular waveguide. Effects of MNP concentration (2.5–20 mg/mL) and microwave frequency (2.0, 2.45 and 2.6 GHz) were evaluated. Further tests with 10 and 20 nm diameter spherical MNPs dispersed within a two-compartment tissue-mimicking phantom were performed with an interstitial dipole antenna radiating 15 W power at 2.45 GHz. Microwave heating of 5 mg/mL MNP-agar phantom mixtures with 10 and 20 nm spherical, and hexagonal MNPs in a waveguide yielded heating rates of 0.78 ± 0.02 °C/s, 0.72 ± 0.01 °C/s and 0.51 ± 0.03 °C/s, respectively, compared to 0.5 ± 0.1 °C/s for control. Greater heating enhancements were observed at 2.0 GHz compared to 2.45 and 2.6 GHz. Heating experiments in two-compartment phantoms with an interstitial dipole antenna demonstrated potential for extending the radial extent of therapeutic heating with 10 and 20 nm diameter spherical MNPs, compared to homogeneous phantoms (i.e., without MNPs). Of the MNPs considered in this study, spherical Fe/Fe_3_O_4_ nanoparticles offer the greatest heating enhancement when exposed to microwave radiation. These nanoparticles show strong potential for enhancing the rate of heating and radial extent of heating during microwave hyperthermia and ablation procedures.

## 1. Introduction

Several clinical trials have demonstrated the benefit of moderate hyperthermia (40 °C < T < 45 °C) adjuvant to radiation and/or chemotherapy for cancer therapy [1,2]. Heating within this temperature range increases tumor blood perfusion, thereby alleviating tumor hypoxia and facilitating improved chemotherapeutic delivery, as well as stimulates a mild anti-tumor immune response [3]. Typically, the goal of the treatment is to raise the temperature of a localized target volume to the desired temperature range, although whole-body hyperthermia has also been investigated [4]. High-temperature thermal ablation (i.e., thermal tissue destruction) involves heating of targeted tissue to temperatures in excess of 55 °C, leading to cell death by coagulative necrosis [5]. Hyperthermic temperatures at the edge of ablation zones offer potential for synergy with other treatment modalities including ionizing radiation, chemotherapeutics, and immunotherapy [6]. Ablation is in clinical use for treatment of tumors in the liver, lung, kidney, bone and other organs. A significant challenge associated with hyperthermia and thermal ablation treatments is the localized delivery of therapeutic energy to minimize thermal damage to healthy tissue, while ensuring adequate thermal dose delivery to the tumor target.

Several non- and minimally invasive energy modalities have been investigated for delivery of hyperthermia, including ultrasound (US) [7,8], radiofrequency (RF) resistive electrodes [9], RF capacitive plates [10], inductive RF systems [11], microwave (MW) antennas [12,13,14] and photothermal therapy [15]. Ultrasound energy offers the ability for precise, non-invasive heating of deep-seated targets [16]; however, it remains technically challenging to target tumors in the lung and behind bones, due to the lack of an appropriate acoustic window [17]. Loco-regional electromagnetic hyperthermia may be delivered with non-invasive [18,19] or minimally invasive interstitial applicators [20]. While non-invasive radiative RF hyperthermia devices offer the ability to heat large target volumes, the limited penetration depth and long electromagnetic wavelength at ~100 MHz limits the specificity of heating deep-seated targets [21]. Interstitial RF electrodes have been widely used for thermal ablation of tumor targets in various organs, but suffer from high treatment variability for tumors greater than 3 cm in diameter [22]. In comparison, interstitial microwave antennas offer the ability to treat larger tumor sizes, faster ablation times, and afford the simultaneous use of multiple applicators [23]. A challenge with heating large tumors using interstitial applicators remains the inadequate heating of cells on the periphery of large tumors, which has been hypothesized to be a cause of the relatively large tumor recurrence rates following thermal tumor ablation. Approaches that enhance the volume of tissue that can be heated to therapeutic temperatures may reduce the variability of thermal therapy procedures.

One approach for increasing the treatment volume with interstitial applicators is the use of contrast agents or materials that afford increased absorption of deposited energy, thereby leading to increased heating. Nanoparticle contrast agents are particularly attractive, due to the potential for selective delivery to the tumors via passive and active targeting strategies [24]. Elliott et al. [25] investigated the use of 120 nm gold nanoshells to enhance heating of tissue-mimicking phantoms when exposed to laser interstitial thermal therapy. They observed heating to greater temperatures in phantoms containing gold nanoshells, as well as a steeper thermal gradient at the boundary between phantom regions with/without nanoshells. Mashal et al. [26] measured the broadband (0.6–20 GHz) complex dielectric properties of tissue-mimicking (TM) phantoms mixed with single-walled carbon nanotubes (SWCNT). The increased dielectric losses afforded by SWCNTs lead to greater heating when exposed to 3 GHz microwaves, compared to phantoms without any SWCNTs. Sun et al. [27] reported on increased in vitro heating of PC-3 human prostate cancer cells when cultured with fullerene (C60) encapsulated within Pluronic F127–chitosan nanoparticles.

Iron oxide nanoparticles are the focus of significant technical and clinical investigation for use in magnetic resonance imaging (MRI) contrast agents [28], magnetic hyperthermia [29,30,31], drug delivery [32,33] and other applications [34]. While the heating of magnetic nanoparticles (MNPs) with alternating magnetic fields has been extensively characterized at ~10 kHz–1 MHz for hyperthermia applications, the electromagnetic properties of magnetic nanoparticles have not been characterized at microwave frequencies (100 MHz–30 GHz). Experimental studies for non-biomedical applications have demonstrated the enhancement of microwave absorption in materials mixed with iron oxide nanoparticles. The optimal characteristics of nanoparticles and electromagnetic exposure parameters that optimize microwave heating are not well understood. In contrast to magnetic nanoparticle hyperthermia, the proposed approach employs nanoparticles to enhance tissue heating, rather than using the nanoparticles as the dominant mechanism for coupling external energy to tissue. Reports have showed that MNPs of different shapes possess different shape anisotropy, which strongly influences the MNP’s heating efficiency in alternating magnetic fields [35]. For example, Bauer et al. have demonstrated that with similar magnetic volume, iron oxide nanocubes heat more efficiently than iron oxide nanospheres [36]. In addition, nanoscale iron oxide particle with different morphologies, such as nano-octopods, nanorods and nanoflowers were reported to show higher specific absorption rate (SAR) values than their spherical counterparts due to their enhanced shape anisotropy [37]. Here we aim to evaluate the effect of MNP shape on microwave heating.

The objective of this study was to assess the feasibility of enhancing microwave heating of tissue-mimicking materials with iron oxide nanoparticle contrast agents. The effects of nanoparticle shape and size on microwave heating enhancements were characterized in a controlled experimental platform. Specifically, 10 and 20 nm diameter spherical Fe/Fe_3_O_4_ nanoparticles, 20 nm edge-length cubic, and 45 nm edge-length/10 nm height hexagonal Fe_3_O_4_ nanoparticles were investigated. Microwave heating enhancements were also evaluated when employing interstitial microwave antennas, similar to those used for thermal ablation and hyperthermia applications.

## 2. Materials and Methods

### 2.1. Preparation of Core/Shell Spherical Fe/Fe_3_O_4_ Nanoparticles

**Figure 1:**
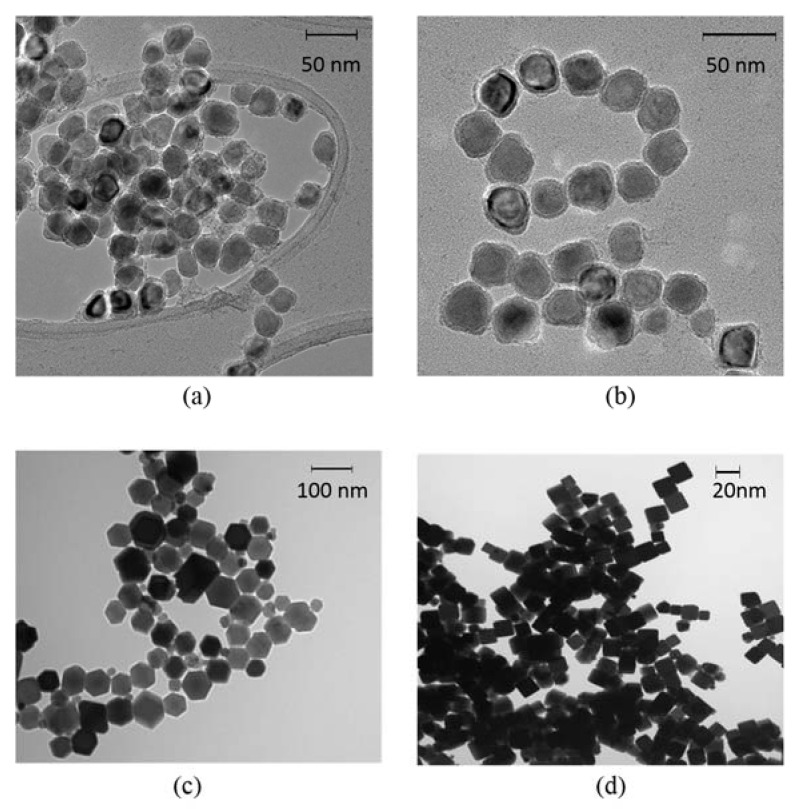
Transmission electron microscopy (TEM) images of the respective magnetic nanoparticles (MNPs) used in this study: (**a**) 10 and (**b**) 20 nm diameter spherical Fe/Fe_3_O_4_, respectively, (**c**) 45 nm edge-length and 10 nm height hexagonal Fe_3_O_4_, and (**d**) 20 nm edge-length cubic Fe_3_O_4_.

Iron nanoparticles were prepared with a slight modification of an established procedure described by Lacroix et al. [38]. A 250 mL, three-necked, round-bottom flask equipped with a magnetic stir bar, one cold water-cooled jacket condenser on the middle neck, one septum and one temperature probe on each of the outer necks was charged with 60 mL 1-octadecene (ODE), 0.9 mL oleylamine and 0.831 g hexadecylammonium chloride (HAD^.^HCl). The reaction system was connected to a Schlenk line through the top of the jacket condenser. The reaction mixture was degassed at 120 °C for 30 min with vigorous stirring. After refilling with argon, the reaction mixture was heated to 180 °C. 2.1 mL Fe(CO)_5_ was injected into the reaction mixture via a syringe over 2 min. The reaction mixture was kept at 180 °C for another 30 min, and cooled to room temperature naturally. The supernatant was decanted, and the iron nanoparticles accumulated on the magnetic stir bar were washed with hexane and ethanol. This method gives 10 nm diameter spherical Fe/Fe_3_O_4_ particles. The 20 nm diameter spherical Fe/Fe_3_O_4_ particles were synthesized by injection of 3.8 mL of Fe(CO)_5_ at the same conditions with those for synthesizing 10 nm diameter particles. The products were dried in vacuum and stored at room temperature for further use. Based on iron, the yields for both reactions were greater than 90%. Transmission electron microscopy (TEM) images of the 10 nm and 20 nm diameter Fe/Fe_3_O_4_ are shown in Figure 1. The spherical Fe/Fe_3_O_4_ samples shown in Figure 1 are all crystalline (X-ray diffraction; XRD), and have a polydispersity of 1.1.

### 2.2. Preparation of Hexagonal and Cubic Fe_3_O_4_ Nanoparticles

A quantity of 0.71 g of Fe(acac)_3_ was added to a mixture of 1.27 g of oleic acid and 0.5 g stearic acid in 10.4 g of benzyl ether. After degassing at room temperature for 1 h, the reaction mixture was heated to 290 °C at the rate of 20 °C/min with vigorous stirring. The reaction mixture was maintained at 290 °C for 30 min, and then cooled to room temperature naturally. The resulted mixture was diluted with 10 mL hexane and 30 mL toluene. The nanoparticles were collected by centrifugation and further washed with chloroform [39]. In the absence of stearic acid under the same conditions, only Fe_3_O_4_ nanocubes were obtained [40]. TEM images of the hexagonal and cubic nanoparticles are shown in Figure 1. The cubic nanoparticles have a polydispersity of 1.1, and the hexagonal nanoparticles have a polydispersity of 1.20.

The nanoparticles considered in this study have previously been extensively characterized in several reports [41,42,43,44].

### 2.3. Experimental Waveguide Testbed

**Figure 2:**
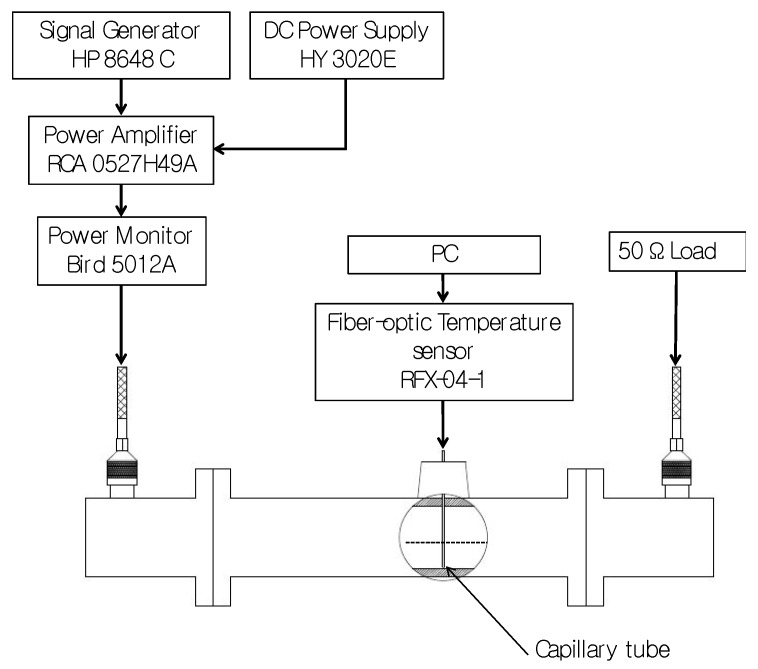
Experimental set up used to characterize microwave heating of MNPs in a rectangular waveguide. DC: Direct Current

To evaluate microwave heating enhancements afforded by candidate MNPs, a custom electromagnetic testbed was implemented to expose MNPs to microwave radiation in a controlled manner (see Figure 2), similar to the setup described in [26]. MNPs were distributed within a gel phantom, and injected into a glass capillary tube (Internal diameter (I.D.) = 1.33 mm, Kimble 34505-99, Rockwood, TN, USA). A fiber-optic temperature probe (Neoptix RFX-04-1, Quebec, Canada) with sampling rate set to 5 Hz was inserted into the capillary tube and the gel was allowed to set around the probe. The capillary tube was positioned within a WR-340 waveguide, through a hole drilled into the center of the waveguide’s broadside. Two coaxial to waveguide transitions, tuned to ensure a reflection coefficient (*S*_11_) ≤ −20 dB in the 2.0–2.6 GHz frequency range, were connected to both ends of the waveguide as shown in Figure 2. Introducing MNP samples within the waveguide yielded minimal changes in the measured reflection and transmission coefficients. MNP samples within the tissue-mimicking gel were exposed to 15 W for 3 min at 2.0 GHz, 2.45 GHz and 2.6 GHz. This power level was selected to constrain peak temperatures within the tissue-mimicking phantom below 70 °C, and thereby limit irreversible changes in the phantom due to microwave heating. To ensure a single-pass exposure of the MNPs to the injected microwave signal, the coaxial to waveguide transition connected to the waveguide output port was connected to a matched load (i.e., 50 Ω). Measured transient temperature profiles were used to calculate the rate of heating during the first 10 s of temperature data immediately after the signal generator was turned off [37].

### 2.4. Magnetic Nanoparticles Test Groups

Heating rates for each candidate MNP dissolved within agar phantoms were measured in the WR-340 waveguide. A 2% agarose water-based phantom was prepared and the candidate MNPs were put into solution at concentrations, by weight, of 20 mg/mL, 10 mg/mL, 5 mg/mL and 2.5 mg/mL (2%, 1%, 0.5%, 0.25%, respectively). Solutions were sonicated to ensure uniform dispersion of the MNPs in the phantom. For each candidate MNP, heating experiments were performed at all four concentrations, when exposed to 15 W, 2.45 GHz microwave radiation with power on for 3 min and off for 1 min. To consider the impact of frequency of the microwave radiation on the measured heating rate, experiments at 10 mg/mL concentration were performed with 15 W input power at 2.0, 2.45 GHz and 2.6 GHz with power on for 3 min and off for 1 min. For all tests, an agarose gel with a 0.1% by weight dopamine solution was used as the control. Experiments were repeated five times for each candidate MNP. Following each experimental measurement, the capillary tube was discarded and replaced with a fresh sample.

For each group from the waveguide experiments, two sample *t*-tests were conducted to test the hypothesis that the rate of heating for the test group was greater than the control group (no MNPs). The significance level was set at 0.05. For the experiments with the interstitial antenna in the two-compartment phantom, two sample *t*-tests were conducted to test the hypothesis that the maximum temperature attained at each radial position (5, 10, 15 or 20 mm from the antenna) was greater than the control group (no MNPs).

### 2.5. Contributions of E- and H-Fields to Microwave Heating

**Figure 3:**
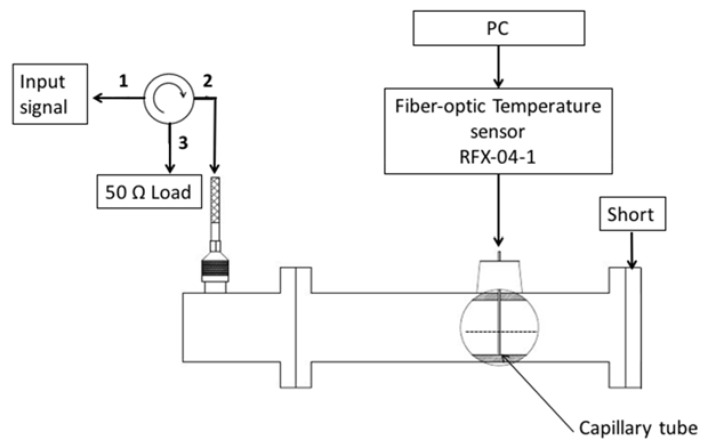
Experimental setup to induce a standing wave within the waveguide, yielding locations with E- and H-field maxima.

Since propagating electromagnetic waves contain both electric and magnetic field components, the experimental platform illustrated in Figure 2 does not provide a means for distinguishing the relative contributions of the E- and H-field components of the electromagnetic wave to the observed heating. To distinguish between heating produced by electric and magnetic fields, we performed further experiments using the adapted circuit shown in Figure 3. By replacing the terminating matched load (50 Ω) with an electrical short, a standing wave is set up within the waveguide. The standing wave has regions of maximum/minimum E- and H-fields. The circulator is employed to direct power reflected back from the waveguide towards a 50 Ω load, thereby protecting the amplifier from large reflected power. Control phantoms, and phantoms with 20 nm spherical MNPs were then positioned at locations corresponding to E- and H-field maxima, as determined from electromagnetic simulations. Specifically, simulations revealed that E-field and H-field maxima were staggered ~43.5 mm apart. In experiments, samples were positioned at 24 mm (H-field maximum) and 71 mm (E-field maximum) along the central axis of the waveguide.

### 2.6. Microwave Heating Enhancements with Practical Interstitial Applicators

**Figure 4:**
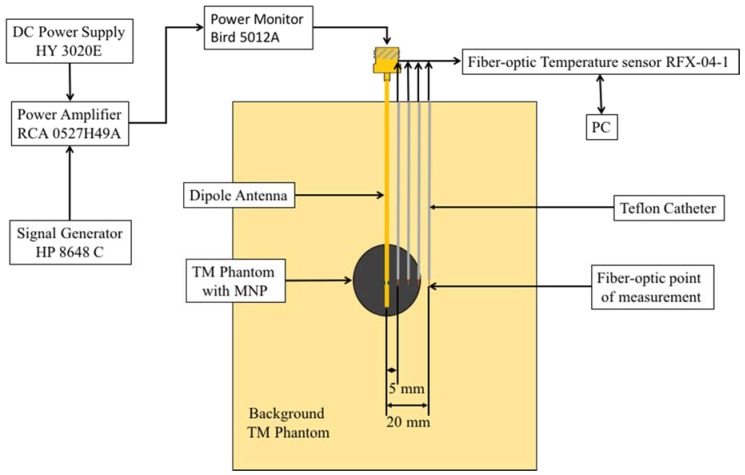
Two-compartment model to characterize microwave heating enhancements with an interstitial dipole antenna. The outer compartment consists of a tissue-mimicking (TM) phantom and the inner compartment is a 30 mm diameter sphere of TM with a 10mg/mL concentration of 10 or 20 nm diameter spherical MNPs.

While the waveguide measurements enable comparison of the MNPs heating enhancements, the generated microwave radiation pattern and small sample volume inside the capillary tube are not representative of practical clinical situations. For this reason, and to confirm that measured heating enhancements were not limited by the geometry of the experimental setup, additional microwave heating experiments were performed with interstitial dipole antennas, similar to those that may be employed for microwave hyperthermia [45]. Figure 4 shows a two-compartment phantom to simulate heating of a tumor containing MNPs with an interstitial microwave antenna. The background TM phantom exhibited electrical properties similar to liver at 2.45 GHz and was created using a procedure similar to the phantom presented in [46] and was set in a 100 mm by 100 mm by 130 mm block. The other compartment was a 15 mm radius sphere created using the TM phantom with a 10 mg/mL MNPs concentration and was placed in the center TM phantom block. The TM phantoms’ electric characteristics were measured at 2.45 GHz using a HP 85070A dielectric probe and compared to measured values of liver tissue. A coaxial dipole antenna was designed to resonate at an operating frequency of 2.45 GHz and was positioned into the center of the 15 mm radius spherical compartment. Temperature was measured, using fiber-optic probes, radially positioned 5 mm, 10 mm, 15 mm and 20 mm from the center of the dipole antenna. For this experiment, 10 nm and 20 nm diameter spherical Fe/Fe_3_O_4_ were employed. Temperature profiles in phantoms with MNPs were compared to profiles measured during microwave heating of a homogenous phantom (i.e., without MNPs). Heating experiments were repeated five times for each test group.

## 3. Results

**Figure 5:**
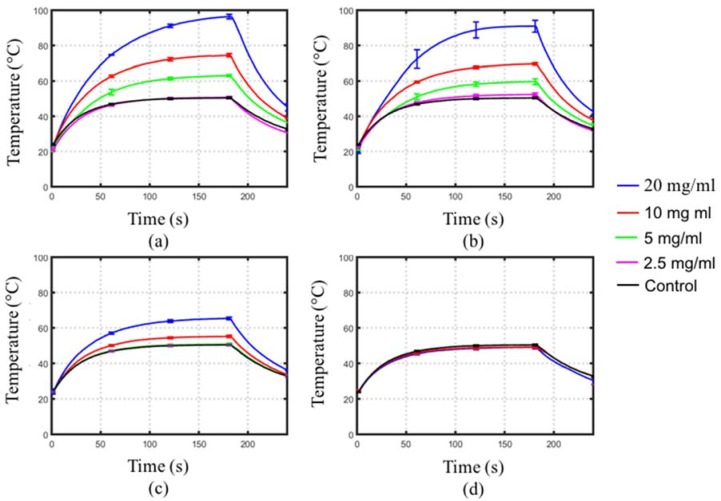
Measured transient temperature profiles of MNPs dispersed within agar at varying concentrations. MNPs considered were: (**a**) 10 nm and (**b**) 20 nm diameter spherical Fe/Fe_3_O_4,_ (**c**) 45 nm edge-length/10 nm height hexagonal Fe_3_O_4_, and (**d**) 20 nm edge-length cubic MNPs. Each curve represents the average of five experiments.

Figure 5 depicts the measured transient temperature profiles during 2.45 GHz microwave radiation of the MNPs mixed within phantoms at different concentrations. Table 1 summarizes the rates of heating for the temperature profiles shown in Figure 5. The heating rates of the phantoms with cubic Fe_3_O_4_ MNPs were lower than control phantoms, indicating a decrease in absorption due to the water-based agar being more electromagnetically lossy than the cubic Fe_3_O_4_ MNPs. For phantoms with hexagonal MNPs, a statistically significant increased rate of heating compared to control was observed only at a concentration of 20 mg/mL. Phantoms with 10 and 20 nm diameter spherical Fe/Fe_3_O_4_ MNPs showed a greater rate of heating compared to control at concentrations of 5, 10 and 20 mg/mL (*p* < 0.05). Furthermore, larger rates of heating were observed in phantoms with 10 nm diameter spherical Fe/Fe_3_O_4_ compared to phantoms with 20 nm diameter MNPs at the same concentration. Overall, higher concentration of MNPs lead to greater heating enhancements.

**Figure 6:**
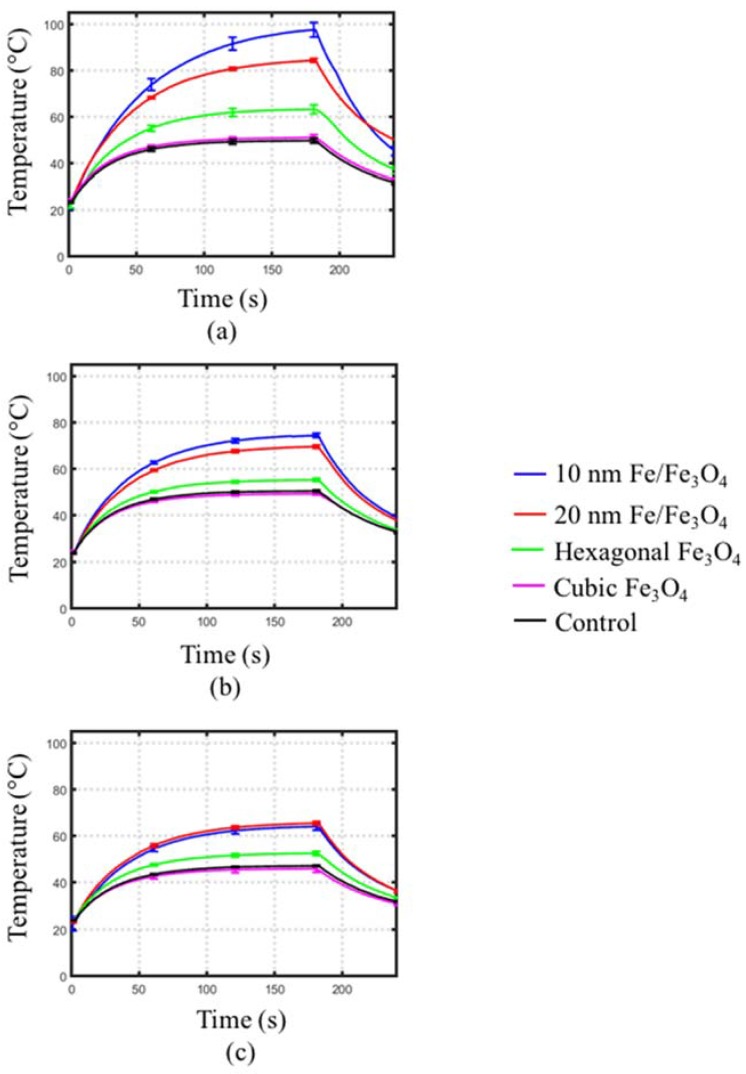
Transient temperature profiles measured in MNP-agar mixtures within the rectangular waveguide at (**a**) 2.0 GHz, (**b**) 2.45 GHz and (**c**) 2.6 GHz. Each curve represents the average of five experiments.

Figure 6 depicts the effect of the frequency of microwave radiation (2.0, 2.45 and 2.6 GHz) on observed transient temperature profiles for cubic and hexagonal Fe_3_O_4_ and for 10 nm and 20 nm diameter spherical Fe/Fe_3_O_4_ at a 10 mg/mL concentration. Table 2 summarizes the rate of heating for the temperature profiles shown in Figure 6. Both spherically- and hexagonally-structured MNP solutions exhibited lower heating rates at 2.45 GHz and 2.6 GHz, compared to 2.0 GHz. Phantoms mixed with 10 nm spherical Fe/Fe_3_O_4_ showed a greater rate of heating (1.7 °C/s) at 2.0 GHz, compared to phantoms with 20 nm spherical Fe/Fe_3_O_4_ (1.23 °C/s). Across all frequencies considered, statistically significant increases in rates of heating were observed in phantoms mixed with spherical MNPs compared to control. For phantoms with hexagonal MNPs, significant increases in heating rates were observed only at 2.0 and 2.6 GHz. There was no increase in rate of heating compared to control for phantoms mixed with cubic MNPs across all frequencies.

**Figure 7:**
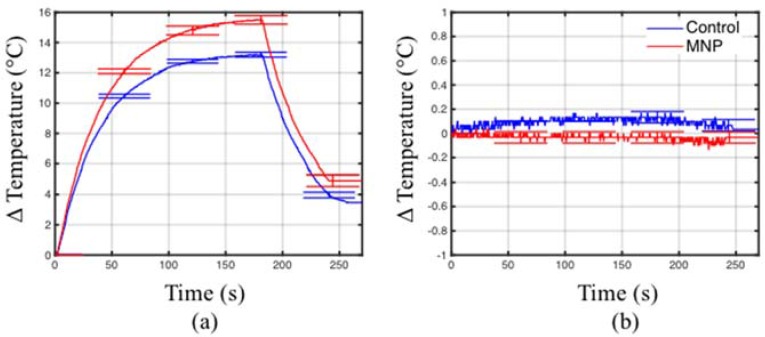
Temperature measured at locations with (**a**) E-field maximum and (**b**) H-field maximum.

Figure 7 shows that no appreciable heating was observed when both control and MNP samples are positioned at locations of the H-field maxima. However, for both samples, heating was observed when positioned at the location of the E-field maxima. This suggests that the dominant mechanism of heating is due to the electric field component.

**Figure 8:**
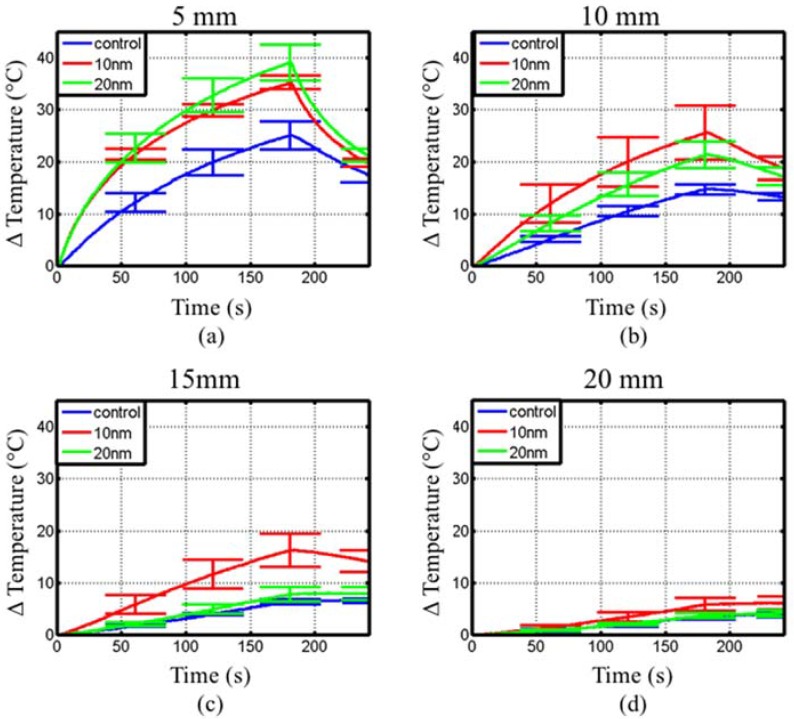
Measured transient temperature profiles within the two-compartment phantom radiated with a 2.45 GHz interstitial dipole antenna. Temperature measurements are shown at (**a**) 5 mm, (**b**) 10 mm, (**c**) 15 mm and (**d**) 20 mm from the antenna. Each curve represents the average of five experiments.

Figure 8 shows the measured temperature rise with fiber-optic probes positioned 5 mm, 10 mm, 15 mm and 20 mm away from a coaxial interstitial dipole antenna powered with 15 W at 2.45 GHz as shown in Figure 4. The tissue-mimicking phantom had a measured dielectric constant, *ε_r_*, of 48 and conductivity, *σ*, of 1.5 S/m at 2.45 GHz, comparable to measured values of liver tissue [47]. Table 3 summarizes the maximum temperature at each position shown in Figure 8. At 5 mm from the dipole antenna, samples with both 10 nm and 20 nm diameter spherical Fe/Fe_3_O_4_ MNPs showed an increase in the initial heating rate and maximum temperature, but were indistinguishable from each other. However, phantoms with 10 nm diameter spherical Fe/Fe_3_O_4_ MNPs yielded a larger maximum temperature and initial heating slope at 10, 15 and 20 mm from the antenna, compared to phantoms with 20 nm diameter spherical MNPs.

**Figure 9:**
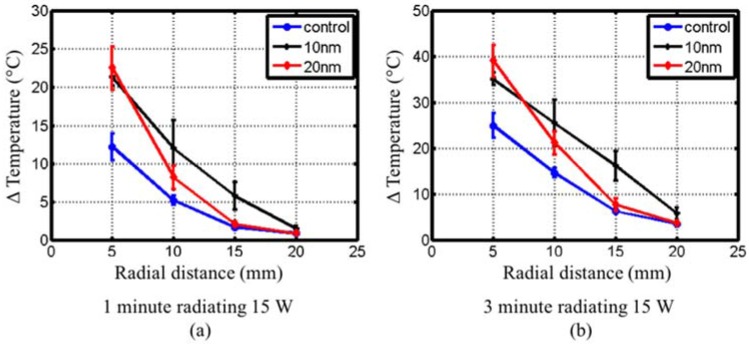
Experimentally measured radial temperature plots at (**a**) 1 min and (**b**) 3 min within the two-compartment phantom.

Figure 9 shows the radial temperature profile for control, 10 nm and 20 nm diameter spherical Fe/Fe_3_O_4_ phantoms after 1 min and 3 min microwave heating. The phantom with 10 nm diameter spherical Fe/Fe_3_O_4_ MNPs yielded a linear decrease with respect to radial distance, while the phantom with 20 nm diameter spherical MNPs and control phantom exhibited a steeper decrease in temperature with respect to radial distance.

## 4. Discussion

Interstitial microwave antennas are in clinical use for minimally invasive thermal ablation and hyperthermia of tumors in various organs. As with other interstitial tissue heating modalities, the radial extent to which tumors can be treated with microwave antennas is limited by the absorption and penetration depth of electromagnetic in tissue and vascular heat sinks. It has been hypothesized that the relatively large tumor recurrence rates following thermal ablation of large tumors, may be in part due to inadequate ablation at the tumor periphery [48]. Therefore, approaches that enable an increased volume of therapeutic heating are desirable, as they may improve the efficacy of heat as a therapeutic modality. The objective of this study was to characterize heating enhancements offered by magnetic nanoparticles, of varying size and shape, when mixed in tissue-mimicking phantoms and exposed to microwave radiation.

Experiments within a rectangular waveguide enabled characterization of transient temperature profiles within phantom-nanoparticle mixtures when exposed to 2.0–2.6 GHz radiation. These experiments revealed that spherical Fe/Fe_3_O_4_ MNPs yielded significant heating enhancements over control, with minimal enhancements offered by cubic and hexagonal MNPs. Statistically significant heating enhancements with spherical MNPs (compared to control) were observed at concentrations of 5 mg/mL and greater. Since previous studies have indicated the potential of surface coating of nanoparticles to contribute to electromagnetic absorption, control experiments included 0.1% by weight dopamine within the phantom. Clinical studies of magnetic fluid hyperthermia with ferrofluids administered by direct injection employed ~4–8% of MNPs to target volume, by weight [49]. Thus, these results suggest that significant heating enhancements may be anticipated with MNP concentrations achieved by direct injection.

Theoretical considerations indicate that electromagnetic absorption increases with increasing frequency, due to larger tissue electrical conductivity at higher frequencies [50]. However, our measurements within the waveguide testbed indicated greater heating enhancements at 2.0 GHz, compared to 2.45 GHz and 2.6 GHz. Electromagnetic power deposition in samples is governed by the complex electrical and magnetic properties of the mixture. Measurements of the broadband electromagnetic properties of the MNPs mixed within TM phantoms should be conducted to validate the heating enhancements observed in this study.

Experiments with interstitial microwave antennas were conducted to determine the extent of microwave heating enhancement feasible with practical antennas, similar to those that may be used within clinical settings. Phantoms for these experiments included two compartments—a central 30 mm diameter sphere of tissue-mimicking phantom mixed with MNP, suspended within a background of tissue-mimicking phantom. This setup was chosen to mimic a tumor loaded with MNPs. Similar to waveguide experiments, phantoms with 10 nm and 20 nm diameter spherical Fe/Fe_3_O_4_ yielded larger heating rates and peak temperatures at distances of 5, 10 and 15 mm, as shown in Figure 8 and Table 3. At a radial distance 20 mm from the dipole antenna, phantoms with 10 nm diameter spherical MNPs only showed a slight rise in temperature compared to control, even though a 5× increase was observed at a distance of 15 mm. This indicates that significant enhancement of heating is restricted to regions with MNPs. After 60 s heating (Figure 9a), both the 10 nm and 20 nm diameter spherical MNP groups yielded a similar temperature at 5 mm from the antenna. However, at 10 mm and 15 mm, temperatures with the 10 nm diameter spherical MNP group were greater than the 20 nm group. At 180 s, the temperature at 15 mm for the 10 nm diameter group was ~10 °C higher compared to the 20 nm and control groups (Figure 9b). Considering an initial tissue temperature of 37 °C in vivo, and an ablative temperature threshold of ~55 °C, a ~18 °C temperature rise is required to induce coagulative necrosis. Our experiments in phantoms indicate the ~18 °C temperature rise threshold extends out to 15 mm for the 10 nm diameter group at 3 min, compared to ~11 mm for the 20 nm diameter group, and ~9 mm for the control. Furthermore, the radial temperature profile for the 10 nm diameter group appears to be approximately linear, compared to an exponential decay for the control and 20 nm diameter groups. This shallower slope suggests a greater role of thermal conduction in the heat distribution. These results indicate that the MNPs may enhance both microwave absorption as well as thermal conduction [25]. Thus, 10 nm diameter spherical MNPs may afford greater peak temperatures and extend the radial extents of therapeutic heating during interstitial thermal therapy procedures.

Previous studies have investigated nanoparticles for enhancing microwave power deposition within tissue-mimicking media to evaluate their potential for improving the efficacy of microwave hyperthermia [26,51]. The results presented here indicate significant heating enhancements offered by 10 nm and 20 nm diameter spherical Fe/Fe_3_O_4_ MNPs similar to that reported with SWCNTs in [26]. However, previous studies did not evaluate microwave heating with practical sources, similar to those used clinically. This study provides experimental evidence indicating that both enhanced electromagnetic absorption and thermal conduction may increase the radial extents of tissue that can be heated to therapeutic levels with interstitial sources.

A limitation of this study is that all considered spherical MNPs were core/shell Fe/Fe_3_O_4_, whereas cubic and hexagonal MNPs were Fe_3_O_4_. Thus, it was not possible to attribute differences in heating rate to only shape or composition. To further elucidate the mechanism of enhanced microwave heating with MNPs, future studies are warranted to characterize the complex electromagnetic and thermal properties of the MNPs embedded within tissue-mimicking materials. Knowledge of MNP electromagnetic and thermal properties may facilitate the design optimization of MNPs that maximize microwave heating enhancements at a desired frequency. This study only considered homogenous distributions of MNPs within tissue-mimicking phantom, which may not be representative of distributions that are achieved in vivo. Further investigations characterizing heating enhancements in an in vivo animal model are warranted to determine heating enhancements feasible with practical MNP distributions in experimental tumors.

## 5. Conclusions

Microwave heating enhancements with spherical Fe/Fe_3_O_4_, hexagonal Fe_3_O_4_, and cubic Fe_3_O_4_ MNPs mixed within tissue-mimicking phantoms were experimentally evaluated. At similar concentrations, spherical Fe/Fe_3_O_4_ MNPs showed greater thermal enhancement than hexagonal Fe_3_O_4_ MNPs. Cubic Fe_3_O_4_ MNPs showed no heating difference when compared to control. Furthermore, it was shown that heating enhancement was greater at 2.0 GHz compared to 2.45 GHz and 2.6 GHz for 10 mg/mL concentration of MNP solutions. It was also shown that MNPs have the potential to improve the radial extents of therapeutic heating with practical interstitial hyperthermia antennas. Further studies will need to be performed to characterize the thermal and electromagnetic properties of MNPs to elucidate the mechanisms of microwave heating enhancements, and facilitate the design optimization of optimal MNPs.

## Figures and Tables

**Table 1:** Heating rate of MNPs mixed within agar: effect of MNP concentration on 2.45 GHz microwave heating.

Sample	MNP Concentration (mg/mL)	Heating Rate (°C/s)	*p*-Value
Cubic	20	0.3 ± 0.03	0.9955
	10	0.3 ± 0.01	0.9946
Hexagonal	20	0.81 ± 0.02	0.0009
	10	0.58 ± 0.02	0.0745
	5	0.51 ± 0.03	0.4197
10 nm spherical	20	1.61 ± 0.03	<0.0001
	10	1.04 ± 0.02	<0.0001
	5	0.78 ± 0.02	0.0015
	2.5	0.53 ± 0.01	0.2702
20 nm spherical	20	1.38 ± 0.1	<0.0001
	10	0.92 ± 0.01	0.0003
	5	0.72 ± 0.01	0.0038
	2.5	0.56 ± 0.02	0.1269
Control	0	0.5 ± 0.1	

**Table 2:** Maximum temperature and heating rate of MNPs mixed within agar: effect of microwave frequency on heating (concentration = 10 mg/mL).

Structure	Frequency (GHz)	Heating Rate (°C/s)	*p*-Value
Cubic	2.0	0.38 ± 0.02	>0.9999
	2.45	0.3 ± 0.01	0.9946
	2.6	0.4 ± 0.01	0.9237
Hexagonal	2.0	0.81 ± 0.08	0.0002
	2.45	0.58 ± 0.02	0.0745
	2.6	0.48 ± 0.01	<0.0001
10 nm Spherical	2.0	1.7 ± 0.04	<0.0001
	2.45	1.04 ± 0.02	<0.0001
	2.6	0.77 ± 0.02	<0.0001
20 nm Spherical	2.0	1.23 ± 0.01	<0.0001
	2.45	0.92 ± 0.01	0.0003
	2.6	0.84 ± 0.01	<0.0001
Control	2.0	0.47 ± 0.02	
	2.45	0.5 ± 0.1	
	2.6	0.41 ± 0.01	

**Table 3:** Heating rates observed at 5, 10, 15 and 20 mm from a 2.45 GHz interstitial dipole antenna inserted in a two-compartment phantom.

Structure	Distance (mm)	Average Temperature Rise (°C)	*p*-Value
10 nm Spherical	5	35.2 ± 1.3	0.0002
	10	25.5 ± 5.1	0.0041
	15	16.2 ± 3.2	0.001
	20	5.8 ± 1.3	0.0077
20 nm Spherical	5	39.02 ± 3.4	<0.0001
	10	21.2 ± 2.6	0.0016
	15	7.74 ± 1.4	0.0413
	20	3.7 ± 0.4	0.3828
Control	5	25 ± 2.7	
	10	14.7 ± 1	
	15	6.3 ± 0.5	
	20	3.6 ± 0.6	
